# Supplementary material for: Semi-quantitative group testing for efficient and accurate qPCR screening of pathogens with a wide range of loads
Source: BMC Bioinformatics. 2024 May 17;25:195. doi: 10.1186/s12859-024-05798-3 (PMC11100062; doi:10.1186/s12859-024-05798-3)
Supplement: Supplementary file 1 — Additional file 1. The Supplementary Information file includes a supplementary figure showing results from error-free PCR simulations and the formal analysis performed in this study. [file 12859_2024_5798_MOESM1_ESM.pdf]

1 Supplementary Information

1.1 Supplementary Figures

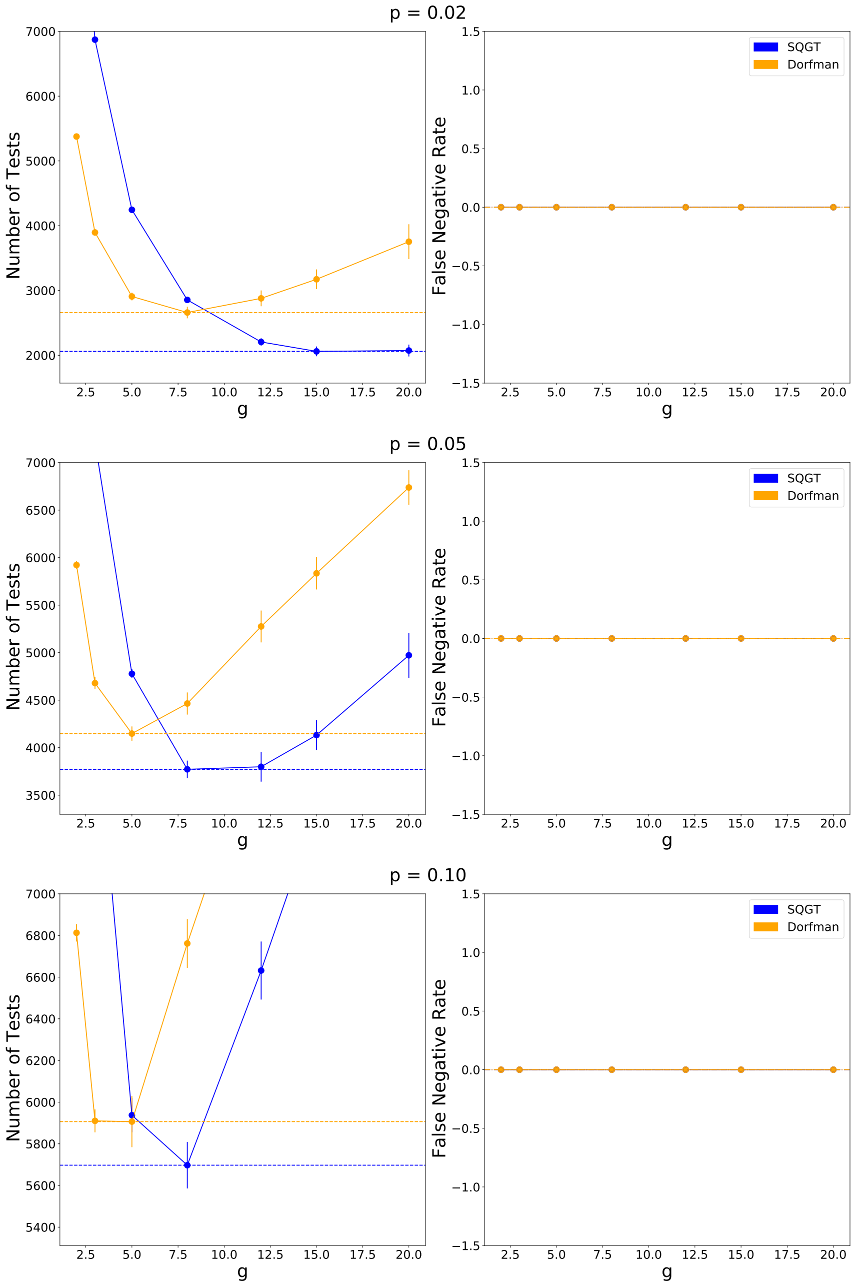

Supplement Figure 1. The number of tests used and the false negative rates using error-free PCR of SQGT (blue), Dorfman's GT (orange) and individual testing (red) for infection rates $p \in \{0.02, 0.05, 0.1\}$. The dashed line marks indicate the number of tests and false negative rates for the optimal group size (where the number of tests is minimized) for each scheme.

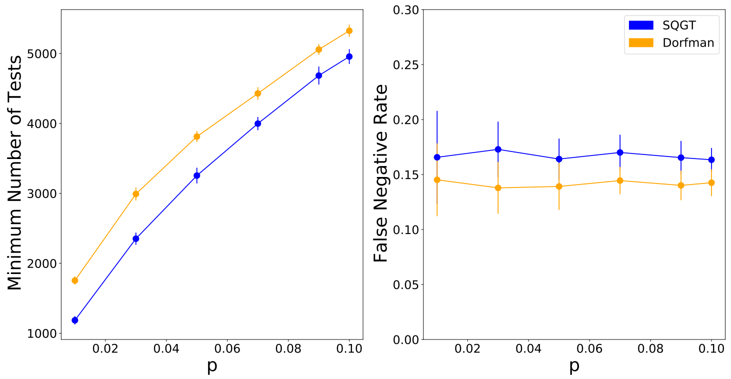

Supplement Figure 2. Evaluating GT schemes using viral loads from a separate data source [10]. The left panel shows the optimal number of tests used in Dorfman's GT (orange) and SQGT (blue) versus the infection rate, p and the right panel shows the corresponding FNRs.

1.2 Analysis of GT models

Group testing (GT) schemes based on binary test outcomes, positive (1) or negative (0), and using single [4] or multiple rounds [2] of pooling can be mathematically analyzed in a straightforward manner. Once again, we consider a population of n individuals and denote the fraction of infected individuals by p. We also assume that the test results are accurate, i.e., the FNR and FPR are identically zero.

For the single-pooling Dorfman's GT, we randomly split the n individuals into groups of size g. Let $E[T/n]$ be the expected number of tests performed per individual under this scheme. In the first stage, we use n/g tests, resulting in $1/g$ tests per individual. In the second stage, individuals in groups with a positive outcome are tested individually, and the rest are declared as "not infected". A group test outcome is positive if at least one infected individual is among the g individuals in the group, which occurs with a probability of $1-(1-p)^g$. Therefore the expected number of tests per individual in the second stage is

$$0*(1-p)^g+1*(1-(1-p)^g)=1-(1-p)^g,$$

and the expected number of tests per individual for the entire scheme is

$$E[T/n]=\frac{1}{g}+1-(1-p)^g.$$

We can perform a similar analysis for a double-pooling scheme. We group the individuals into groups of size g such that each individual contributes to two different groups. This can either be achieved via two random permutations of the list of individuals, similar to SQGT, or via a 2D array of wells with appropriately chosen dimensions, as described in [2]. Therefore, for each individual, we have a pair of test outcomes. If both the test outcomes are positive (1), we test individually otherwise we declare the individual as not infected.

In the first stage, we use $2n/g$ tests, resulting in $2/g$ tests per individual. In the second stage, an individual is tested if both group tests are positive. We consider two scenarios: the individual is infected with probability p, or the individual is not infected with probability $1-p$. If the individual is infected, both groups they contributed to will test positive, leading to testing in the second stage. If the individual is not infected, the group test outcome is positive if and only if at least one other person in the group is infected. For the individual to get tested in the second stage, this condition must hold for both groups they are part of in the first stage. This allows us to calculate the expected number of tests in the second stage as

$$1*(p+(1-p)(1-(1-p)^{g-1})^2).$$

Therefore, the expected number of tests per individual is

$$E[T/n] = 2/g + p + (1-p)(1-(1-p)^{g-1})^2.$$

This approach can be extended to a multi-pooling strategy, with each individual contributing to more than two group tests. However, such schemes have diminishing returns in the number of tests saved due to substantial overlaps in the groups [2].

Our Semi-Quantitative Group Testing (SQGT) scheme extends the double-pooling strategy by utilizing semi-quantitative information from a qPCR test. This scheme improves performance by avoiding the direct individual testing of low and medium-risk individuals. Under the assumption that the FNR of qPCR testing is zero, our relaxed SQGT scheme (see Results) yields the same results as double-pooling.

1.3 Probabilistic SQGT with variable viral load

We analyze how the semi-quantitative scheme performs when infected individuals may have either low or high viral loads. This is relevant to account for heavy hitters, individuals with substantially higher viral loads which can mask infected individuals with low viral loads. To this end, we consider a simplified model where each individual is independently infected and presents a low viral load at the time of testing with probability p_{i1}, or is infected and presents a high viral load at the time of testing with probability p_{i2}. In particular, each individual is infected (regardless of their viral load) with total infection probability $p = p_{i1} + p_{i2} < 1$.

Individuals with high viral loads are problematic because, based on the semi-quantitative output of qPCR, groups featuring *one* such individual may be mistaken for groups with *several* infected individuals with low-to-intermediate viral loads.[1] This phenomenon naturally leads us to consider the following modified version of testing: A test applied to a group of individuals has outcome 0 if there are no infected individuals in the group, outcome 1 if there exists *exactly* one infected individual with *low* viral load, and 2 if either there exists more than one infected individual with low viral load or at least one infected individual with *high* viral load. Therefore, as expected, individuals with high viral load obfuscate the test outcomes.

We assume that the population contains n individuals, each of which is independently positive with some probability $p = p_{i1} + p_{i2} < 1$, as explained. In the first stage, we divide the n individuals into groups of size g. The groups are denoted by $\gamma_1, \gamma_2, \ldots, \gamma_{n/g}$. In the second stage, we proceed as follows:

- If a pool γ_i tests 0, we declare all individuals in γ_i as negative.
- If a pool γ_i tests 1, we apply a nearly-optimal zero-error nonadaptive GT scheme to detect the infected individual.
- If a pool γ_i tests 2, we test all individuals in γ_i separately.

We can compute the expected number of tests per individual of the testing scheme, $E[T/n]$, as a function of the probability of infection p and the first-stage pool size g as follows. First, we observe that for the scheme outlined above, we are guaranteed to have *exactly* 1 infected individual in any pool that tested 1. We also know that zero-error nonadaptive GT schemes to detect τ infected individuals in a group of size g can be designed with $m(g,\tau) = c \cdot \tau^2 \log(g/\tau)$ tests for some constant $c > 0$. As a corollary, we know that for detecting one infected individual,

[1] This is not problematic for *binary* GT, where the test outcomes do not distinguish between one or several infected individuals in the group.

$m(g,1) = \lceil \log g \rceil$ tests are needed. This can be achieved by using a Hamming code parity-check matrix. This gives us the expected number of tests as

$$\mathbb{E}[T/n] = \frac{1}{g} + p_1 \cdot \lceil \log g \rceil + p_2, \tag{1}$$

where p_1 and p_2 denote the probability that a given pool tests 1 and 2, respectively.

The probability that a group of size g contains exactly one infected individual with low viral load and zero individuals with high viral load (leading to test outcome 1) is

$$p_1 = g \cdot p_{i1} \cdot (1 - p_{i1} - p_{i2})^{g-1} = g \cdot p_{i1} \cdot (1-p)^{g-1},$$

while the probability that the group contains either more than one infected individual with low viral load or at least one individual with high viral load (leading to test outcome 2) is

$$p_2 = 1 - g \cdot p_{i1} \cdot (1 - p_{i1} - p_{i2})^{g-1} - (1 - p_{i1} - p_{i2})^g = 1 - g \cdot p_{i1} \cdot (1-p)^{g-1} - (1-p)^g.$$

Combining these observations, we conclude that the expected number of tests per individual as a function of p_{i1} and p_{i2} is given by

$$\frac{1}{g} + g \cdot p_{i1} \cdot (1-p)^{g-1} \cdot \lceil \log g \rceil + 1 - g \cdot p_{i1} \cdot (1-p)^{g-1} - (1-p)^g, \tag{2}$$

where $p = p_{i1} + p_{i2}$.

For fixed p_{i1} and p_{i2}, it is easy to numerically minimize the expression above as a function of g to find the optimal group size for the scheme under consideration. On the other hand, the expected number of tests per individual for the basic Dorfman's GT [5] is

$$\frac{1}{g} + 1 - (1-p)^g, \tag{3}$$

and the expected number of tests per individual for a double-pooling scheme with binary tests [2, 3] is

$$\frac{2}{g} + p + (1-p)(1-(1-p)^{g-1})^2. \tag{4}$$

Figures 3 and 4 compare the expected number of tests per individual required by various schemes for different values of the total infection probability p and the specific infection probabilities p_{i1} and p_{i2}. Clearly, double pooling outperforms Dorfman's single pooling GT strategy, while semi-quantitative testing with single pooling outperforms both single and double pooling in the expected number of tests. SQGT combines the ideas of double pooling and semi-quantitative information from tests to obtain further savings in test results. We do not have a closed-form expression for SQGT due to the complexity of the scheme. However, as reported in the Results, double pooling SQGT provides substantial savings over Dorfman's GT (single pooling) while maintaining low FNR for real-world GT data.

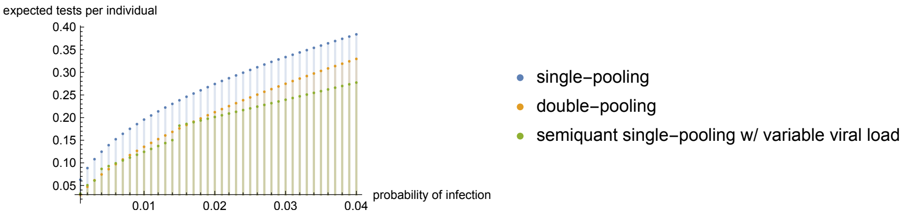

Supplement Figure 3. Comparison between the expected number of tests per individual required by Dorfman's single pooling scheme [5], the double pooling scheme [2, 3], and our semi-quantitative single pooling scheme as a function of total infection probability p with $p_{i1} = 0.84p$, $p_{i2} = 0.16p$.

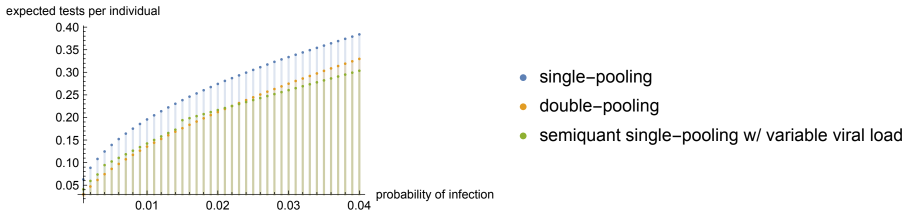

Supplement Figure 4. Comparison between the expected number of tests per individual required by Dorfman's single pooling scheme [5], the double pooling scheme [2, 3], and our semi-quantitative single pooling scheme as a function of total infection probability p with $p_{i1} = 2p/3$ and $p_{i2} = p/3$.

1.4 Lower bounds for nonadaptive probabilistic SQGT

The main text discusses how to replace the second stage of SQGT with nonadaptive GT. Designing nonadaptive GT schemes reduces to identifying test-matrices that satisfy disjointness or separability properties [6]. One such scheme involves sampling a random binary matrix such that every entry is an i.i.d. Bernoulli(q) random variable with $0 < q < 1$. Each row of this matrix defines a pool for group tests. Given sufficiently many rows, this matrix represents a zero-error nonadaptive GT scheme with high probability.

We, therefore, focus on deriving a theoretical result that establishes lower bounds for nonadaptive probabilistic GT that may be used to assess the quality of our adaptive schemes. For this purpose, we adapt an argument by Aldridge [1] for arbitrarily small error probability under a constant probability of infection. More precisely, we consider a setting where each test has $m + 1$ outcomes for some $m \geqslant 1$: The outcome of a test is either i if there are exactly i infected individuals for $i < m$, and $\geqslant m$ otherwise. This corresponds to the setting introduced in [7] which provides the most informative type of measurements one can expect from the SQGT framework using the amplification curve information. This model accounts for the saturation limit for each test, dictated by m, which is a phenomenon observable from the amplification curve. Moreover, as before we assume that each individual in the population of size n is infected independently with some constant probability $p > 0$. We show the following.

Theorem 1 *For every m and constant $p > 0$ there exists a constant $\epsilon(m, p) > 0$ such that, under the setting described above, nonadaptive testing requires at least n/m tests to achieve error probability less than $\epsilon(m, p)$ in a population of size n.*

In contrast, for $m = 2$, our two-stage scheme uses significantly fewer than $n/2$ tests provided p is not very large.

Proving Theorem 1 follows by a simple adaptation of an approach by Aldridge [1], who showed that individual testing is required in order to achieve arbitrarily small error in regular nonadaptive probabilistic GT (which corresponds to $m = 1$). First, given any nonadaptive testing scheme, we may without loss of generality remove all tests with m or fewer elements, along with all individuals who participate in those tests. This does not affect the lower bound. Then, we show that there are no nonadaptive testing schemes with an arbitrarily small error where every test includes at least $m + 1$ individuals. Combining these two observations immediately yields Theorem 1.

For an individual i, let x_i denote its infection status. Call an individual i (regardless of its infection status) *disguised* if every test t in which it participates contains at least m other individuals which are infected. If i is disguised, then changing x_i from 0 to 1, or vice-versa, does not change the outcome of the testing scheme. As a result, we can do no better than guess x_i, and we will be wrong with probability at least $\min(p, 1 - p)$. To finalize the argument, it suffices to show there is a disguised individual with constant probability.

Let D_i denote the event that individual i is disguised, and let $D_{t,i}$ denote the event that individual i is disguised in test t. Since the $D_{t,i}$ are increasing events[2], the Fortuin-Kasteleyn-

[2]If $D_{t,i}$ holds and the set of infected individuals is expanded, then $D_{t,i}$ continues to hold under this expanded set.

Ginibre (FKG) inequality [8] implies that

$$\Pr[D_i] \geqslant \prod_{t:x_{t,i}=1} \Pr[D_{t,i}], \tag{5}$$

where $x_{t,i}$ indicates whether individual i participates in test t. Moreover, we have

$$\Pr[D_{t,i}] = \Pr[B(w_t - 1, p) \geqslant r], \tag{6}$$

where $w_t = \sum_{i=1}^{n} x_{t,i}$ is the weight of test t and $B(w_t - 1, p)$ denotes a binomial random variable with $w_t - 1$ trials and success probability p.

Let

$$L_i = \log\left(\prod_{t:x_{t,i}=1} \Pr[D_{t,i}]\right) = \sum_{t:x_{t,i}=1} \log \Pr[D_{t,i}] = \sum_{t=1}^{T} x_{t,i} \log \Pr[D_{t,i}],$$

where T denotes the total number of tests, which we assume satisfies $T/n < 1$, and log denotes $\log_2$. Then, it suffices to show that there exists some $i^\star$ with $L_{i^\star} > c$ for some constant c independent of n. Let I be uniformly distributed over $\{1, 2, \ldots, n\}$, and let $\overline{L} = \mathbb{E}[L_I]$. We have

$$\begin{aligned}
\overline{L} &= \frac{1}{n} \sum_{i=1}^{n} \sum_{t=1}^{T} x_{t,i} \log \Pr[D_{t,i}] \\
&= \frac{1}{n} \sum_{t=1}^{T} w_t \log \Pr[D_{t,i}] \\
&\geqslant \min_{t=1,\ldots,T} w_t \log \Pr[B(w_t - 1, p) \geqslant m] \\
&\geqslant \min_{w \geqslant r+1} w \log \Pr[B(w-1, p) \geqslant r] =: L^\star,
\end{aligned}$$

where the second equality follows from the fact that $\Pr[D_{t,i}]$ is the same for every i such that $x_{t,i} = 1$, and in the first inequality we use the assumption that $T/n < 1$. It is immediate that there exists some $i^\star$ with $L_{i^\star} \geqslant \overline{L}$, which implies that $\Pr[D_{i^\star}] \geqslant 2^{L^\star}$. Therefore, the error probability of the testing scheme is at least $\epsilon(m, p) = \min(p, 1-p) \cdot 2^{L^\star}$. Noting that $L^\star$ does not depend on n and is bounded from below for any m and p concludes the proof (since $\lim_{w\to\infty} w \log \Pr[B(w-1, p) \geqslant m] = 1$).

1.5 Extension of Hwang's model [9] to SQGT

Definition of $TPR_1(p, g)$. We can define the conditional probability $TPR_1(p, g)$ following the same idea as in Hwang's paper as

$$\begin{aligned}
TPR_1(p, g) &= \mathbb{P}(\text{test score is 1}|\text{there is exactly 1 positive subject in the group}) \\
&= \frac{A(p, g)}{g \cdot p \cdot (1-p)^{g-1}},
\end{aligned} \tag{7}$$

where $A(p, g)$ is chosen such that $TPR_1(k)$ satisfies the following two limit conditions given the infection rate $p < 0.5$:

$$TPR_1(p, 1) = 1, TPR_1(p, \infty) = 0. \tag{8}$$

Specifically, $TPR_1(p, \infty) = 0$ holds since there will be only 1 infection in this group of size infinity. Based on (8), one simple form of $A(p, g)$ can be $A(k) = p^g$, which implies

$$TPR_1(p, g) = \frac{p^g}{g \cdot p \cdot (1-p)^{g-1}}. \tag{9}$$

When $g = 1, TPR_1(p,1) = 1$; when $g \to \infty$, $TPR_1(p,g) = \frac{1}{g}(\frac{p}{1-p})^{g-1}$. Since we assume $\frac{p}{1-p} < 1$, so $TPR_1(p,\infty) \to 0$.

When taking the dilution effect into consideration, we introduce the coefficient d as in [9]. When $d = 0$, there is no dilution effect, meaning that $TPR_1(p,g) = 1$ for every choice of group size g; when $d = 1$, the dilution is complete and the probability should be of the form (9). Therefore, the final expression for $TPR_1(p,g)$ with dilution effects would be

$$TPR_1(p,g,d) = \frac{p^{g^d}}{g^d \cdot p \cdot (1-p)^{g^d-1}}. \tag{10}$$

Definition of $TPR_2(p,g)$. We can define the conditional probability $TPR_2(p,g)$ as

$$\begin{aligned} TPR_2(p,g) &= \mathbb{P}(\text{test score is 1 or 2}|\text{there are at least 2 positive subjects in the group}) \\ &= \frac{B(k)}{1-(1-p)^g - g \cdot p \cdot (1-p)^{g-1}}. \end{aligned} \tag{11}$$

The two limit conditions are

$$TPR_2(p,2) = 1, TPR_2(p,\infty) = p. \tag{12}$$

It is worth pointing out that the limiting of $TPR_2(p,\infty)$ is very different from $TPR_1(p,\infty)$, since now there can be many infections in this huge group, so the limiting probability will be approximately the probability of sampling a subject from the population uniformly at random and the subject being positive. In this case, we can have

$$TPR_2(p,g) = \frac{p^{1+2/g}}{1-(1-p)^g - g \cdot p \cdot (1-p)^{g-1}}. \tag{13}$$

It is easy to check that the limits (12) hold for $TPR_2(p,g)$.

When taking the dilution effect into consideration, we can again make use of the dilution coefficient d. The final expression for $TPR_2(p,g,d)$ would be

$$TPR_2(p,g,d) = \frac{p^{1+(g/2)^{-d}}}{1-(1-p)^{2(g/2)^d} - 2(g/2)^d \cdot p \cdot (1-p)^{2(g/2)^d-1}}. \tag{14}$$

Expected cost. With $TPR_1(p,g,d)$ and $TPR_2(p,g,d)$, we can compute the expected cost of one round of SQGT test. The expected number of infections in a group, when there is only 1 infection in the group, is always 1. Meanwhile, the expected number of infections in a group, when there are at least 2 infections in the group and the group size is g, is

$$\begin{aligned} \mathbb{E}(\text{infections}|\text{\# of infections} \geqslant 2) &= \sum_{i=2}^{g} i \cdot \mathbb{P}(\text{\# of infections = } i|\text{\# of infections} \geqslant 2) \\ &= \frac{\sum_{i=2}^{g} i \cdot \mathbb{P}(\text{\# of infections = } i)}{\mathbb{P}(\text{\# of infections} \geqslant 2)}. \end{aligned}$$

Note that $\sum_{i=0}^{g} i \cdot \mathbb{P}(\text{\# of infections = } i) = pg$ based on the binomial distribution, so $\sum_{i=2}^{g} i \cdot \mathbb{P}(\text{\# of infections = } i) = pg - pg(1-p)^{g-1} = pg(1-(1-p)^{g-1})$. We have

$$\mathbb{E}(\text{infections}|\text{\# of infections} \geqslant 2) = \frac{pg(1-(1-p)^{g-1})}{1-(1-p)^g - pg(1-p)^{g-1}}. \tag{15}$$

So the total expected cost is

$$\begin{aligned}E_{\text{cost}}(g) &= \frac{n}{g} \cdot (\text{expected cost for each group of size } g) \\ &= \frac{n}{g} \cdot \big\{1 + pg(1-p)^{g-1}\,(TPR_1(p,g,d) \cdot g + (1 - TPR_1(p,g,d)) \cdot c) + \\ &\quad (1-(1-p)^g - pg(1-p)^{g-1})(TPR_2(p,g,d) \cdot g + \\ &\quad (1 - TPR_2(p,g,d)) \cdot c \cdot \mathbb{E}(\text{infections}|\#\text{ of infections} \geqslant 2))\big\},\end{aligned}$$

where c is the cost if we misidentify a positive group, and for simplicity set the lab cost for each test to \$1. By taking the derivative of $E_{\text{cost}}(g)$ to 0 we can get the optimal group size g in this case.

References

1. M. Aldridge. Individual testing is optimal for nonadaptive group testing in the linear regime. *IEEE Transactions on Information Theory*, 65(4):2058–2061, 2019.

2. T. Berger and J. W. Mandell. Bounds on the efficiency of two-stage group testing. In *Codes, Curves, and Signals: Common Threads in Communications*, pages 213–232. Springer, 1998.

3. A. Z. Broder and R. Kumar. A note on double pooling tests. *arXiv e-prints*, Apr. 2020.

4. R. Dorfman. The detection of defective members of large populations. *The Annals of mathematical statistics*, 14(4):436–440, 1943.

5. R. Dorfman. The detection of defective members of large populations. *The Annals of Mathematical Statistics*, 14(4):436–440, 1943.

6. D. Du, F. K. Hwang, and F. Hwang. *Combinatorial group testing and its applications*, volume 12. World Scientific, 2000.

7. A. Dyachkov and V. Rykov. Generalized superimposed codes and their application to random multiple access. In *Proc. 6th Int. Symp. Inf. Theory*, volume 1, pages 62–64, 1984.

8. C. M. Fortuin, P. W. Kasteleyn, and J. Ginibre. Correlation inequalities on some partially ordered sets. *Communications in Mathematical Physics*, 22(2):89–103, 1971.

9. F. K. Hwang. Group testing with a dilution effect. *Biometrika*, 63(3):671–680, 1976.

10. T. C. Jones, G. Biele, B. Mühlemann, T. Veith, J. Schneider, J. Beheim-Schwarzbach, T. Bleicker, J. Tesch, M. L. Schmidt, L. E. Sander, et al. Estimating infectiousness throughout sars-cov-2 infection course. *Science*, 373(6551):eabi5273, 2021.
